# Pathogenetic Mechanisms of T Cell Dysfunction in Chronic HBV Infection and Related Therapeutic Approaches

**DOI:** 10.3389/fimmu.2020.00849

**Published:** 2020-05-12

**Authors:** Paola Fisicaro, Valeria Barili, Marzia Rossi, Ilaria Montali, Andrea Vecchi, Greta Acerbi, Diletta Laccabue, Alessandra Zecca, Amalia Penna, Gabriele Missale, Carlo Ferrari, Carolina Boni

**Affiliations:** ^1^Laboratory of Viral Immunopathology, Unit of Infectious Diseases and Hepatology, Azienda Ospedaliero-Universitaria di Parma, Parma, Italy; ^2^Department of Medicine and Surgery, University of Parma, Parma, Italy

**Keywords:** chronic HBV infection, T cell exhaustion, immune-therapy, liver environment, immunoregulatory mechanisms

## Abstract

A great effort of research has been devoted in the last few years to developing new anti-HBV therapies of finite duration that also provide effective sustained control of virus replication and antigen production. Among the potential therapeutic strategies, immune-modulation represents a promising option to cure HBV infection and the adaptive immune response is a rational target for novel therapeutic interventions, in consideration of the key role played by T cells in the control of virus infections. HBV-specific T cells are severely dysfunctional in chronic HBV infection as a result of several inhibitory mechanisms which are simultaneously active within the chronically inflamed liver. Indeed, the liver is a tolerogenic organ harboring different non-parenchymal cell populations which can serve as antigen presenting cells (APC) but are poorly efficient in effector T cell priming, with propensity to induce T cell tolerance rather than T cell activation, because of a poor expression of co-stimulatory molecules, up-regulation of the co-inhibitory ligands PD-L1 and PD-L2 upon IFN stimulation, and production of immune regulatory cytokines, such as IL10 and TGF-β. They include resident dendritic cells (DCs), comprising myeloid and plasmacytoid DCs, liver sinusoidal endothelial cells (LSECs), Kupffer cells (KCs), hepatic stellate cells (HSCs) as well as the hepatocytes themselves. Additional regulatory mechanisms which contribute to T cell attrition in the chronically infected liver are the high levels of soluble mediators, such as arginase, indoleamine 2,3-dioxygenase (IDO) and suppressive cytokines, the up-regulation of inhibitory checkpoint receptor/ligand pairs, the expansion of regulatory cells, such as CD4+FOXp3+ Treg cells, myeloid-derived suppressor cells and NK cells. This review will deal with the interactions between immune cells and liver environment discussing the different mechanisms which contribute to T cell dysfunction in chronic hepatitis B, some of which are specifically activated in HBV infection and others which are instead common to chronic inflammatory liver diseases in general. Therapeutic interventions targeting dysregulated pathways and cellular functions will be also delineated.

## Introduction

Chronic HBV infections remain a major public health problem worldwide (1). Currently, there are no curative treatments and available therapies are effective in inhibiting HBV replication, but are of limited efficacy on cccDNA and HBsAg concentrations, thereby requiring long-lasting administrations to avoid the risk of HBV reactivation at withdrawal (2–4). In the search for more effective therapies, possible candidates are compounds with direct anti-viral or immune modulatory activity. The latter strategy is supported by the evidence of dysfunctional innate and adaptive immune responses in chronic active hepatitis B (CHB), which contribute to HBV persistence (5, 6). Recent studies unveiled a number of altered regulatory mechanisms which are key for the impairment of anti-viral immune responses in chronic infections, involving different cellular populations of the immune system, suppressive soluble mediators, up-regulation of co-inhibitory molecules (7, 8). Most of these inhibitory mechanisms take place within the liver which is a tolerogenic organ that must prevent excessive immune responses against pathogens and antigens derived from the gut, to protect the host against severe immune-mediated damage (8). The tolerogenic properties of the liver are further enhanced by chronic inflammation which can trigger several regulatory mechanisms that make T cell exhaustion in HBV infection particularly severe, allowing HBV to acquire survival advantage over the immune system and to persist in the infected host.

This review will elucidate the relationship between adaptive anti-viral immune responses and the different virus- and host-related mechanisms particularly active within the chronically inflamed liver which favor HBV persistence. We will focus on: (i) T cell dysfunction, including up-regulation of co-inhibitory signaling pathways, metabolic alterations, apoptotic cell death and phenotypic/functional heterogeneity of HBV-specific T cells; (ii) the effect of the persistent exposure of immune cells to high antigen loads; (iii) the features of the liver environment, comprising tolerogenic antigen presenting cells (APC), suppressive soluble factors, local induction of suppressive regulatory cells; (iv) potential immune-therapeutic strategies based on functional T cell reconstitution.

## T Cell Exhaustion in Hepatitis B

**FIGURE 1:**
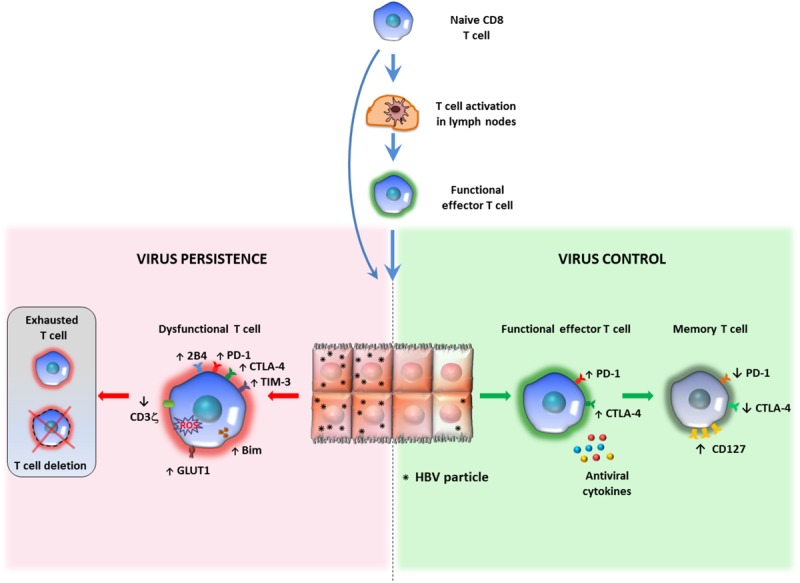
Relationship between antigen persistence and anti-viral T cell responses in the liver. Priming of naïve CD8 T cells can occur in lymph nodes (LNs) or within the liver. In the lymph nodes naïve CD8 T cells differentiate into functional effectors; after migration to the liver, if the majority of hepatocytes are infected and express high antigen levels, virus-specific CD8 T cells undergo functional impairment or physical deletion. Some specific features of exhausted HBV-specific CD8 T cells are the over-expression of multiple inhibitory receptors and pro-apoptotic molecules, CD3ζ chain down-regulation and various metabolic alterations (*left panel*). If only a minor proportion of liver cells express limited amounts of viral antigens, virus specific CD8 cells can maintain efficient anti-viral activity and can promote virus control and antigen clearance (*right panel*). This model of T cell activation derives form studies performed in mouse models of HBV infection, but no definitive evidence is available in human infection to confirm that induction of functionally efficient effector CD8 T cells is driven by the amount of antigen (number of infected hepatocytes and amount of antigen expressed by individual liver cells) and that decline of antigen can allow restoration of functionally efficient HBV-specific CD8 T cell responses.

During chronic HBV infections virus-specific T-cells appear deeply exhausted (5). Both CD8 and CD4 T cells, up-regulate co-inhibitory receptors which can inhibit the T cell function upon cross linking of their corresponding ligands (9–16). Overexpression of such co-inhibitory molecules was originally described in exhausted T cells from LCMV-chronically infected mice (17, 18), although up-regulation of inhibitory receptors is known to play a physiological role in the contraction of effector acute phase responses to avoid excessive immune pathology and autoimmune disorders. Indeed, during acute, self-limited HBV infection activated functional effector T cells display high PD-1 levels, as expression of activation, which tend to decrease during the recovery phase (9, 19–22) (Figure 1). However, in the setting of virus persistence, chronic antigen stimulation leads to the sustained expression of inhibitory receptors in association with T cell dysfunction (23). Liver-infiltrating HBV-specific T cells show maximal up-regulation of PD-1 and to a lesser extent of other co-inhibitory receptors, such as 2B4, LAG3, and CD160 (10, 11, 24) (Figure 1). On the other hand, overexpression of several checkpoint ligands, such as PD-L1 and galectin-9, has been observed on circulating and intrahepatic antigen-presenting cells and on liver resident Kupffer cells (KCs), respectively (14, 25, 26). Mechanistically, PD-1 engagement causes the de-phosphorylation of the costimulatory receptor CD28 and of other TCR-associated components, thereby leading to the attenuation of the corresponding signaling and to up-regulation of inhibitory genes (27–30). Moreover, PD-1 and CTLA-4 signalings also intervene in T cell metabolism, by inhibiting glycolysis (31).

Exhausted HBV-specific T cells have also been depicted as more prone to apoptosis, mediated by the up-regulation of the death receptor TRAIL-2 and the pro-apoptotic mediator BIM (32–34). They harbor dysfunctional mitochondria with an abnormally elevated ROS content, have a poor capacity to use oxidative phosphorylation but are instead strictly dependent on glycolysis to meet cell energy demands (35, 36) (Figure 1). The relevance of these metabolic defects to T cell exhaustion is confirmed by the effect of mitochondria-targeted anti-oxidant compounds in the reconstitution of the anti-viral T cell function *in vitro* (36).

As described for CD8 T cells in cancer and in other viral infections, also HBV-specific CD8 T cells from chronically infected patients are not a functionally homogeneous population of exhausted cells, because distinct T cell subsets with different degrees of dysfunction have been identified (37, 38). Moreover, different levels of exhaustion have been reported for T cell subsets of different HBV antigen specificity. Higher expression of exhaustion markers, associated with a lower expansion capacity has been reported for polymerase-specific compared to core-specific CD8 cells from chronic HBV patients with low viral load (39, 40). Such heterogeneity has been associated to variable levels of sensitivity to functional restoration treatments in other models of T cell exhaustion (41–46). Thus, analysis of CD8 T cell heterogeneity in individual chronic patients is worth being investigated as a possible tool to identify those patient populations that are more likely to respond to immune therapeutic interventions.

Inhibitory checkpoint blockade has been widely studied as a strategy for immune reconstitution in chronic HBV infection. Many *in vitro* studies showed that PD-1/PD-L1 blockade, alone or in combination with the manipulations of other pathways, can induce variable levels of improvement of both T and B cell responses, because high PD-1 levels have also been detected in dysfunctional HBV-specific B cells from chronic HBV patients (9–12, 14, 15, 24, 47–50). A reduction of the pro-apoptotic Bim molecule expression and an increase in cytokine-producing CD8 T cells have been observed upon CTLA-4 blockade (15) and manipulation of the 2B4 and Tim-3 pathways as well (14, 16). All different checkpoint modulation approaches, however, are not free of toxicities or immune-related adverse events, as reported in cancer patients (51–54). Moreover, additional limitations to the *in vivo* use of checkpoint inhibitors is the wide heterogeneity of T cell responses reported *in vitro* to this treatment (11), and the lack of simple predictors to identify with some level of accuracy those patients who could benefit from PD-1 blockade either alone or in association with other costimulatory (for example, CD137 or OX40 stimulation) (10, 48), or co-inhibitory (CTLA-4 or TIM-3) (14, 15), pathway manipulation. The *in vitro* study of HBV-specific T cell functionality cannot be widely used to predict response to therapeutic immune modulation *in vivo* because of its complexity and the need of a better standardization of functional assays. A much simpler possibility, which is being explored in different laboratories, consists in the use of phenotypic panels, including exhaustion and memory molecules, to study total, unfractionated T cells, in view of data indicating that exhaustion can partially affect also the overall CD4 and CD8 T cell populations. In this regard, the downregulation of CD3ζ and CD28 has been associated to functional defects in total non-antigen-specific CD8 T cells *in vitro* and *in vivo* in CHB patients with high viral load (55). Besides, a more recent investigation confirmed the presence of both HBV-specific and global T cell dysfunction mediated by multiple regulatory mechanisms, including overexpression of PD-1 and CTLA-4 by CD4 T cells (47). Moreover, additional studies have highlighted also the unconventional γδ T cell role in HBV pathogenesis both in acutely infected chimpanzees (56) and in acute and chronic patients (57). Despite some controversial findings (58–61), a recent report described innate-like phenotypes based on the expression of Tbet/Eomes, and reduced PD-1, in association with a functional alteration in circulating Vδ2 + γδ T cells during ALT flares in CHB patients (57).

In spite of the multifaceted nature of the immune dysfunction, an improvement of the virus-specific immunity has been detected in nucleoside analog (NUC)-treated chronically infected woodchucks upon the association of PD-L1 blockade with therapeutic DNA vaccination, leading to suppression of viral replication and anti-WHs antibody seroconversion in two out of three animals (62). In another study, a durable control of viremia and antigenemia was induced in 2 of 11 WHV-infected woodchucks when anti-PD-L1 was associated to NUC treatment (63).

In a recent study in human HBV infection a single dose anti-PD-1 (Nivolumab) was administered to NUC treated, virally suppressed HBeAg negative chronic HBV patients who were compared to NUC treated patients who received anti-PD-1 plus therapeutic vaccination. Reduction of HBsAg titers was detected only in a limited proportion of patients who received NUC combined with anti-PD-1, with a total and persistent HBsAg loss in only one of them. Remarkably, no severe adverse events were reported, but, disappointingly, no additional effect was observed on serum HBsAg concentrations as well as on strength and quality of anti-viral T cell responses in the cohort of patients treated also with therapeutic vaccination (64). Low dose and short time administration of anti-PD-1 in this study don’t allow, however, to draw definitive conclusions about possible future applications of this potential treatment.

In summary, *in vitro* data support the concept that only a proportion of patients should partially benefit from check-point blockade because only a limited percentage of patients respond *in vitro* and very rarely functional restoration involves simultaneously all important anti-viral cell-mediated immunological parameters. Moreover, in view of the multi-factorial nature of T cell exhaustion pathogenesis in HBV infection, the possibility to reconstitute T cell functionality with a single intervention selectively focused on immune check-points seems to be highly unlikely.

This conclusion is further reinforced by the recent finding that PD-1 blockade doesn’t allow complete correction of the specific epigenetic profiles which are associated with CD8 T cell exhaustion (65, 66). This finding gives theoretical supports to the concept that inhibition of the PD-1 pathway cannot allow persistent and complete correction of T cell dysfunction in chronic viral infections. Based on our present understanding of HBV-specific T cell exhaustion, PD-1/PD-L1 blockade should be likely seen as a possible adjuvant therapy to be used only in a selected group of HBV infected patients to improve T cell function and antigen responsiveness before the use of more specific HBV antigen-based therapies, such as vaccination with an appropriate antigenic composition. Moreover, the lack of an epigenetic effect provides the rationale for combining checkpoint blockade with epigenetic drugs, a therapeutic strategy already tested in the setting of anti-tumor immunotherapy.

## Persistent T Cell Exposure to High Viral Antigen Concentrations

The amount of antigen expressed by liver cells is believed to influence the fate of effector CD8 cells but available data do not allow to draw definitive conclusions on this issue.

*In vitro* studies have addressed the interplay between infected hepatocytes and anti-viral T cells, showing the strengthening of the T cell function as antigen expression increases, suggesting that high viral antigen production is needed for efficient T-cell activation within the liver (67). On the same line, an antigen dose-dependent anti-viral T cell function increment has been observed also in an infectious HCV-hepatoma cell co-culture model, in which a cognate epitope expression threshold has been investigated, indicating a peptide concentration range for effector T cell activation (68). Also the effector:target ratio has been shown to modulate the HCV-specific T cell function in an HCV replicon system, with non-cytolytic mechanisms prevailing at lower ratio values (69). However, as *in vivo* high antigen loads are associated to co-inhibitory receptor/ligand overexpression, when PD-L1 expressing hepatoma cells were used, recapitulating more closely the liver environment, the T cell cytolytic activity resulted significantly inhibited (68). Indeed, experiments performed in animal models, evidenced a more complex T cell regulation in the liver environment.

By exploiting a recombinant adeno-associated viral vector system (rAAV8 transduction) to obtain selective antigen expression in mouse hepatocytes *in vivo*, a threshold of antigen in the liver was identified by the Bertolino’s group as a crucial factor tuning T cell differentiation. In this model, persistent cytotoxic T lymphocyte (CTL) function was maintained only when less than 25% of hepatocytes were transduced, indicating that low frequencies of antigen-expressing hepatocytes can elicit a functional CD8 T cell response (70, 71). Conversely, when antigen was expressed by a high percentage of hepatocytes, virus-specific CD8 T cells became less responsive and over time underwent T-cell exhaustion and deletion. The phenotypic analysis of intrahepatic T cells isolated from mice treated with high doses of rAAV revealed that T cell exhaustion was associated with high levels of the inhibitory PD-1 and Tim-3 receptors as well as with a deficiency in cytotoxic activity and anti-viral cytokine production (71). Therefore, these data suggest that the level of antigen expression and the proportion of infected hepatocytes represent key factors in driving the development of adaptive T cell responses in chronic viral hepatitis.

These results are consistent with previous findings in a transgenic mouse model where intrahepatic antigen presentation triggered negative regulatory signals leading to a dysfunctional differentiation of naïve CD8+ T lymphocytes. Indeed, naïve HBV-specific CD8 T cells adoptively transferred into transgenic mice with high intrahepatic HBV antigen expression displayed proliferative responses but lacked the capacity to differentiate into functional effector T cells. The mechanism of CD8 T cell dysfunction involved PD-1 signaling and could be rescued by CD40-dependent mDC activation (72, 73).

More recently, intrahepatic T cell differentiation was studied in a HBV transgenic mouse system in which T cell priming was restricted to the liver. In this model, naive CD8+ TCR transgenic T cells specific for HBV core were injected into major urinary protein (MUP)-core transgenic mice which exclusively expressed a non-secretable version of the HBV core protein in 100% of hepatocytes. Naïve T cells primed in the presence of high levels of HBV core antigen expressed proliferative function but failed to differentiate into functionally competent effector cells. Remarkably, even when CD8 T cell priming occurred within a liver environment where HBV core antigen expression, induced by injection with a low dose of a hepatotropic adeno-associated viral vector (AAV) encoding the HBV core protein, was limited to less than 5% of hepatocytes, effector differentiation was not supported despite much lower levels of antigen expression (74). These different T cell differentiation fates observed in different experimental models may likely be related not only to variable thresholds of antigen expression within the liver in different infection/transfection models, but may also depend upon TCR affinity which may restrict effector priming to high affinity TCR/MHC interactions. The evidence that a substantial reduction of hepatic antigen expression by more than 15-fold in individual hepatocytes was still insufficient to induce a functionally efficient effector CD8 T cell differentiation, force some caution in predicting what antigen decline induced by therapy can actually do on the T cell function in chronic hepatitis patients. Future experiments in chronic patients treated with anti-viral drugs able to diminish the antigen load are mandatory to address this fundamental issue because no evidence is available at present in natural HBV infection of whether decline of antigen can actually cause functional T cell improvement, and, if so, which magnitude of antigen decline is required for induction of a biological effect. This is particularly puzzling in a clinical setting where multiple factors simultaneously contribute to T cell exhaustion and where individual patients harbor a widely heterogeneous HBV-specific CD8 T cell population comprising cells with different levels of functional impairment and different degrees of affinity for target cell recognition.

In another HBV-transgenic (tg) mouse model of therapeutic vaccination, based on a heterologous vaccination strategy with initial protein priming followed by recombinant Modified Vaccinia Ankara virus (MVA) vector-boost, serum HBV antigen levels have been reported to influence the immunological responsiveness to therapeutic vaccination. Indeed, vaccine-induced HBV-specific CD8+ T cell responses inversely correlated with antigenemia levels before vaccination. In mice with high antigen levels both HBsAg/MVA-S and HBcAg/MVA-core immunization failed to induce envelope and core-specific CD8 T cell responses in the spleen and in the liver, while vaccination in mice with low or intermediate antigen levels allowed expansion of anti-viral T cell responses and control of infection (75). Interestingly, HBV-specific CD8 T-cells were induced efficiently by therapeutic vaccination after RNAi-mediated suppression of hepatic antigen expression in highly antigenemic mice which were non-responsive to antigen stimulation before anti-viral therapy (76). Although very promising, translation of this therapeutic approach to the human natural infection may be not so easy because antigenemia in chronic patients is generally much higher and of longer duration than in this mouse model and because exposure to high antigen loads is only one of the multiple mechanisms of T cell inhibition which are simultaneously in place after decades of infection in chronic hepatitis patients.

In the setting of natural human HBV infection, a hierarchy of T cell functional efficiency was described in different conditions of HBV control in relation to serum HBsAg concentration (77). For example, maximal T cell functional efficiency was observed in the resolution phase of an acute self-limited hepatitis, associated with complete control of infection and lack of HBsAg in the serum, followed by intermediate levels of T cell functionality in chronic inactive carriers with partial control of infection and low levels of HBsAg, and finally maximal impairment of T cell responses in chronic active hepatitis patients with high levels of viremia and antigenemia (77).

**FIGURE 2:**
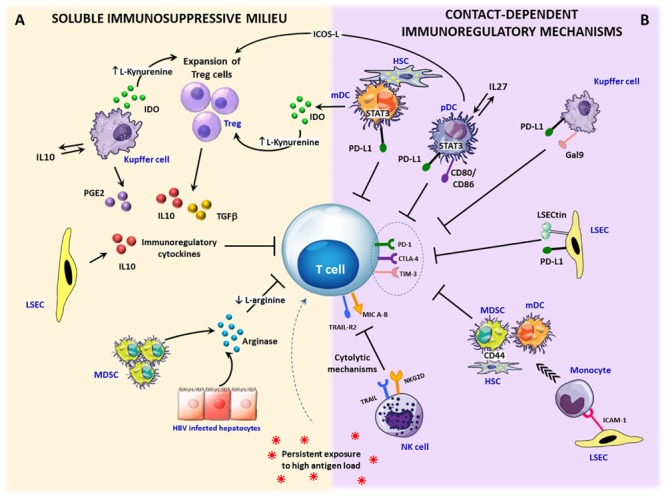
Immunosuppressive circuits in the liver. Within the infected liver, persistent expression of viral antigens in concert with different immunoregulatory pathways drive T cell differentiation toward exhaustion. Upon inflammation, IFN-γ stimulation activates immunosuppressive mechanisms that can be mediated by soluble factors or require cell to cell contact. **(A)** Soluble factors: immunosuppressive cytokines, such as TGF-β and IL-10 can be produced by expanded regulatory T cells (Treg) as well as by stellate cells (HSCs), dendritic cells (DCs) and Kupffer cells (KCs). In addition, a number of liver infiltrating cells, such as monocytes, macrophages and dendritic cells, can release the tryptophan-degrading enzyme indoleamine 2,3-dioxygenase (IDO), which causes either tryptophan depletion with consequent dampening of T cell proliferation and function or the generation of toxic catabolites, such as kynurenines, which can cause T cell apoptosis and CD4 differentiation in Treg cells. Another key soluble mediator is represented by arginase, released by damaged hepatocytes and myeloid-derived suppressor cells (MDSC), inducing arginine depletion. Lack of arginine determines CD3ζ downregulation and suppression of T cells proliferation. Moreover, the enzyme cyclooxygenase-2 produced by activated KCs participates in the synthesis of the immunosuppressive prostaglandin E2 (PGE-2). **(B)** Contact-dependent mechanisms: antigen-specific CD8 T cells can be killed by NK cells through NKG2D- and TRAIL-dependent lysis. Over-expression of inhibitory ligands (e.g., PD-L1, Galectin-9) on liver endothelial sinusoidal cells (LSECs), dendritic cells, KCs, and stellate cells facilitates the triggering of T cell inhibitory pathways. In addition, inflammatory monocytes recruited into the liver through ICAM-1 expression on LSECs can differentiate into myeloid-derived suppressor cells by a CD44-dependent mechanism driven by activated stellate cells. Finally, Treg cell expansion and IL-10 production can be caused by pDCs and mDCs via the ICOS/ICOSL-mediated interaction or IDO secretion.

Although level of antigen and duration of T cell exposure to antigenic epitopes certainly affect T cell functionality and responsiveness to exogenous stimulation, we must consider, however, that the level of functional T cell efficiency is the final result of the interplay between T cells and a wide range of inhibitory mechanisms (Figures 1, 2). Thus, the presence of a high antigen load in chronically infected hosts certainly represents an important obstacle for curative immunotherapeutic approaches but we must be aware that decline of antigen alone is unlikely to be sufficient for successful recovery of protective immune responses because multiple mechanisms contribute simultaneously to T cell exhaustion in chronic HBV infection (78).

## The Liver Environment

A number of inhibitory mechanisms are active within the liver making the intrahepatic environment highly tolerogenic. The unique composition of the hepatic cell population, the expression of inhibitory checkpoint ligands and the presence of soluble regulatory mediators are some of the factors which contribute to the tolerogenic nature of the liver, which is maximal in the presence of chronic inflammation, irrespective of the etiology. Therefore, most of the mechanisms which will be described in this section are common to different chronic inflammatory liver diseases and are not specifically expressed only in chronic HBV infection (Figure 2).

### Tolerogenic APC

Liver APC are not functionally mature as their counterparts in other organs (79). Hepatic dendritic cells (HDCs) and liver resident macrophages, known as KCs, which represent conventional APC, but also non-professional, unconventional APC, such as liver sinusoidal endothelial cells (LSECs), hepatic stellate cells (HSCs), as well as the hepatocytes themselves, contribute to immune tolerance, because they differ significantly from cells present in the circulation or in secondary lymphoid organs (80).

Hepatic dendritic cells show an immature phenotype, with a lower expression of MHC and costimulatory molecules (i.e., CD40, CD86, and CD80), poor endocytotic capacity and low IL-12 production, compared to their peripheral counterparts (81, 82). As reported for HDCs, low level expression of co-stimulatory and MHC class I and II molecules at the steady state makes also KCs, LSECs, and HSCs poorly efficient in T cell stimulation (83–88). They can also induce CD8 T cell apoptosis by Fas-FasL interaction, as described for KCs following reactive-oxygen species (ROS) production induced by FasL up-regulation on their surface (80, 89), and for HSCs through induction of intracellular signaling pathways triggered by PD-L1 and B7-H4 crosslinking (90–93). Hepatocyte priming of CD8 T cells generally results either in clonal T cell deletion by BIM-mediated apoptosis (90, 94), or in dysfunctional CD8 T cells without effector functions (74), as already discussed in detail in the previous section. HSCs and LSECs can also inhibit CD8 T cell priming/activation through a TRAIL and ICAM-dependent mechanism or through LSECtin-CD44 interaction, respectively (95). Moreover, antigen presentation by all these cells is often associated to the secretion of immune-regulatory mediators, such as IL-10, TGF-β, prostaglandin E2 (PGE2), and to the up-regulation of PD-L1 (87, 96–100).

High co-inhibitory molecule expression by HDCs can be induced by the nucleotide-binding oligomerization domain 2 (NOD2) signaling, significantly more expressed in hepatic plasmacytoid (pDCs) than in conventional or myeloid (mDCs) dendritic cells, which is also responsible for their low IFN-α secretion (101). The CD141+ subset of mDCs, which produces IL-12 and plays a central role in antigen cross-presentation (102), have also been reported in healthy human livers to express high levels of the tolerogenic molecules immunoglobulin-like transcript 3 (ILT3) and ILT4 (103), which can inhibit T cell activation through tyrosine-based inhibitory motifs (ITIMs) contained in their cytoplasmic tails (104, 105).

In chronic HBV patients hepatic and peripheral dendritic cells have been reported by several studies to be functionally impaired, with a possible inhibitory effect of viral antigens on their function (106–112). However, this concept is still widely debated (113) because other studies did not observe any significant alterations of the DC function (114, 115).

Among the possible DC defects in chronic hepatitis B, a reduced production of the immunoregulatory cytokine osteopontin (OPN) has been described and suggested to represent a potential cause of TH1 response impairment (116). Impaired DCs maturation and function in chronically HBV-infected patients has also been associated with an altered expression of innate sensors, such as TLRs (117–120).

Although most studies on DCs in chronic HBV infection have been performed on peripheral cells, a recent report shows alterations in frequency and basal activation status of DC subsets from both blood and liver of chronic HBV patients compared to healthy controls. In addition, also a defective up-regulation of maturation markers upon TLRs triggering was reported in this study, but only circulating DCs displayed a significant impairment of cytokine production in response to TLR agonist stimulation (118). The poor effect on the NK cell cytolytic function by TLR9 ligand stimulation of HDCs was related to reduced OX40L expression as well as to high plasma IP-10 and HBV antigen levels (121). Mechanistically, HBV particle internalization can block pDCs IFNα production by inhibiting TLR9 signaling, through down-regulation of TLR9 transcription (122), which can also be indirectly caused by TNFα and IL-10 production induced by HBsAg on monocytes (123).

Also KCs have a role in the induction of tolerance to HBV. This is supported by the observation in a mouse model of HBV infection that earlier HBV clearance in older mice was accompanied by a severe reduction in KC numbers, concomitant with a sharp induction of TNFα-producing Ly6C+ monocytes, followed by the proliferation of IFNγ+TNFα+ CD8 T cells. Instead, younger mice, which failed to clear the virus, maintained high frequencies of IL-10-secreting KCs (124).

Functional skewing of the T cell response toward a TH2 profile can also be supported by macrophages with a predominant M2-like phenotype, known to be potent immune suppressor cells (125), detected at high frequencies in a humanized mouse model of persistent HBV infection (126). The evidence that KCs and liver infiltrating macrophages show up-regulation of the CD86 co-stimulatory molecule, which is known to drive T cell differentiation toward a functional TH2 profile (127), detected by immunohistochemistry staining of liver biopsies from chronic HBV patients, further support this finding (128). A direct interaction between HBsAg and KCs with HBsAg uptake, was documented both *in vitro* and in *ex vivo* isolated KCs from CHB patients (129). *In vitro* exposure to HBV antigens preferably induced TGF-β, rather than pro-inflammatory cytokine secretion by primary rat KCs (130); moreover, HBV antigen interaction with TLR2 on KCs caused T cell inhibition through IL-10 secretion (131, 132) and TLR2 knockout or KC depletion resulted in enhanced HBV elimination and improved CD8+ T cell responses in mice (131). This inhibitory effect mediated by HBV proteins was further documented by more recent studies showing that HBV protein uptake by intrahepatic macrophages from CHB patients can favor anti-inflammatory over pro-inflammatory functions and can ultimately promote hepatocyte infection (133).

Despite these tolerogenic features, the liver environment must ensure a fine tuning of divergent functions to guarantee tolerance to antigens introduced through the gut, but also to initiate efficient immune responses needed for pathogen control. For example, in case of liver inflammation KCs secrete pro-inflammatory cytokines and can mediate full CD8 T cell differentiation (83, 134), while the propensity of hepatic DCs toward immunogenicity or tolerance has been associated to their lipid content and metabolism. Indeed, high lipid content can make HDCs able to secrete high levels of pro-inflammatory cytokines and to efficiently activate T or NK cells, while low lipid content is associated with tolerance induction (135).

Overall, many pieces of evidence indicate a preferential anti-inflammatory response induction by antigen-presenting cells in chronic HBV infection, suggesting the possibility of a direct innate immunity inhibition by the virus itself (119), which has also been reported to be poorly sensed by innate immunity sensors, behaving as a stealth virus (136). Interference of viral proteins with innate signaling pathways (119) represents another theoretical reason why reduction of antigenemia may be relevant in the perspective of therapeutic strategies for CHB based on functional immune reconstitution. In addition to novel direct anti-viral agents acting at different levels of the HBV life cycle (see below in the “Perspectives for novel therapeutic strategies” section), stimulation of pathogen recognition receptors, such as TLRs and Retinoic acid-Inducible Gene I (RIG)-like receptors (137) represents a possible strategy, currently under evaluation, to increase the efficiency of anti-viral immune activation.

Dendritic cells manipulation has also been proposed. Indeed, administration of HBV peptide-loaded DCs was reported to elicit functional HBV-specific CD8 cells and to reduce the viral load in Hepato-HuPBL mice (138) and a targeted antigen delivery to DCs was proven to efficiently induce local immunity to hepatotropic viruses in mice (139). Interestingly, in the context of cancer immunotherapy, PD-L1 silenced DCs were used for a DC vaccine that induced potent T cell responses (140). Finally, IL-2 administration could recover dysfunctional HBV-specific CD8 cells originated by an inefficient hepatocyte priming in HBV transgenic mice (74).

### Soluble Factors

As a result of its peculiar cell population, the liver microenvironment is enriched in soluble factors with immunosuppressive function (Figure 2A). Not only the immunoregulatory cytokines IL-10 and TGF-β are preferentially secreted over other pro-inflammatory cytokines by several types of hepatic cells (i.e., dendritic cells, macrophages/monocytes, LSECs) (141), but also an abundance of other regulatory mediators has been detected within the liver. Indeed, constitutive expression by different liver infiltrating cells (e.g., dendritic cells, regulatory myeloid cells) (141, 142), of tryptophan-2,3-deoxygenase (TDO), or Indoleamine 2,3-dioxygenase (IDO) which are tryptophan-degrading enzymes, can lead to tryptophan depletion and the formation of toxic metabolites, such as kynurenines (143, 144). These factors have been shown to constrain T and NK cell proliferation (145) and to induce T cell apoptosis (143, 144).

The shortage of another essential amino acid, i.e., L-arginine, causing T cell arrest in the G0/G1 phase and CD3ζ chain down-regulation, is due to an excess of arginase derived from damaged hepatocytes (146) and other liver-infiltrating cells (147). In HBV-infected patients the latter mechanism was demonstrated both in acutely and in chronically infected subjects and could be corrected *in vitro* by T cell transfection of CD3ζ or replenishment of the amino acid arginine required for its expression (19, 55).

Interestingly, a granulocytic subset of myeloid-derived suppressor cells (gMDSC), which release arginase I upon degranulation, was demonstrated to be expanded in CHB patients with high HBV replication levels without liver necro-inflammation, highlighting the capacity of these cells to contain tissue damage by limiting arginine supply to T cells (148).

### Local Induction of Regulatory Cells

In addition to HSCs, KCs, and macrophages, the liver is enriched in populations of regulatory cells, such as myeloid derived suppressor cells (MDSCs) and regulatory T cells (Tregs), that can promote tolerance by producing immunosuppressive cytokines (IL-10 and TGF-β) and by expressing high levels of co-inhibitory ligands, such as PD-L1 and Galectin-9 (Figures 2A,B). Importantly, the direct contact between circulating T cells and resident regulatory cells is favored by the slow blood flow in liver sinusoids and by the unique liver architecture, which is characterized by the presence of sinusoidal wall fenestrations which facilitate cell to cell contact (85).

#### Myeloid Derived Suppressor Cells

Myeloid cells play a key regulatory role within the liver contributing to the outcome of immune responses, thanks to their functional plasticity. In fact, they can differentiate from monocytes into macrophages, monocyte-derived dendritic cells or myeloid suppressor cells. During liver inflammation, inflammatory monocytes can be recruited into the liver as a result of ICAM-1 (CD54) expression on LSECs (141). They can subsequently differentiate into myeloid-derived suppressor cells by a CD44-dependent mechanism driven by activated stellate cells (141), as shown by the possibility to prevent acquisition of the suppressive phenotype by blocking CD44-mediated interaction between monocytes and HSCs (149). During prolonged hepatic inflammation, MDSCs inhibit immune responses through different mechanisms, including IL10 and TGF-β secretion, as well as production of arginase 1 and reactive oxygen species (ROS) (150). Expansion and suppressive function of MDSCs was reported to be modulated by cysteine-rich protein 61 (CCN1), a multifunctional protein highly expressed in impaired cholangiocytes and hepatocytes. This has been described in primary biliary cholangitis (PBC) but it may likely represent a general mechanism of MDSC modulation shared by chronic liver inflammations of different etiology (151).

As already mentioned, the immunosuppressive function of MDSCs was described in chronic HBV infection where gMDSC could dampen HBV-specific T cell responses in a arginase-dependent manner, documenting the capacity of expanded arginase-expressing gMDSCs to regulate liver immunopathology (148). A recent study in chronically HBV infected patients with high levels of HBsAg revealed the capacity of MDSCs to promote immune dysfunction through the induction of regulatory T cells, primarily via TGF-β and IL-10 dependent signaling pathways. Interestingly, a year tenofovir treatment did not result in the immune restoration of the regulatory MDSC and Treg populations (152). In addition, circulating MDSCs were significantly expanded in patients with HBV-related acute-on-chronic liver failure (ACLF) and closely associated with disease progression and severity. In this setting, CD3ζ chain expression was decreased in T cells and negatively correlated with MDSC frequency (153).

Monocyte differentiation within the liver can also be driven by TLR-9 signaling toward the acquisition of anti-viral protective properties through the formation of inflammatory monocyte aggregates, called iMATEs, where virus-specific CD8 cells can expand upon OX40- and CD28-mediated signaling initiated by inflammatory dendritic cells of monocyte origin (154). This unique iMATEs structure, which, however, seems to be generated selectively in the mouse liver, can contribute to chronic virus infection control. iMATE formation induced by systemic TLR-9 treatment has also been reported to induce effector CD8 T cell expansion and control of tumor growth in a murine hepatoma model (155).

Such functional plasticity of MDSCs represents a unique challenge for future therapeutic interventions in the setting of chronic liver disease (156). Indeed, all these results suggest that a therapeutic regiment that either blocks MDSC suppressive functions and Treg amplification or supports iMATES formation facilitating CD8 T cell proliferation might represent a potential strategy to cure HBV-related liver disease. However, further studies of this unique regulatory cell population are needed to better characterize its real therapeutic potential.

#### Treg Expansion

During viral infection, regulatory T cells are recruited into the inflamed liver and compete with effector CD8 T cells for IL-2, limiting the amplification of virus-specific T cell responses (8). Both mDCs and pDCs have been described to promote Treg proliferation through a mechanism mediated by STAT3 signaling (102, 157–159). Specifically, the expansion of Treg cells, which can inhibit T cell responses either by direct cell-cell contact or by secretion of suppressive cytokines, can be caused by plasmacytoid dendritic cells (pDCs) through an IL27-based circuit which can lead to PD-L1 expression and subsequent Treg proliferation (158, 160). pDCs further foster T cell tolerance by stimulating IL-10 producing Tregs via an ICOS/ICOSL-mediated interaction (102, 159). In parallel, mDCs can also promote Treg expansion and T cell apoptosis as a result of the cross-talk with HSCs, leading to PD-L1 up-regulation and IDO induction, and subsequent generation of immunosuppressive kynurenine compounds (161–163). Recent findings highlighted a novel OX40L + DC subset able to selectively expand Tregs, which plays an essential role in Treg homeostasis maintenance under inflammatory conditions; indeed, co-culture of DCs from GM-CSF treated mice and CD4+ T cells induced an increase in Treg proliferation in an OX40L dependent manner (164). Treg cell expansion can be also stimulated by LSECs and IFN-γ activated stellate cells in a PD-L1 independent manner (141, 161). LSECs are the major liver cells responsible for TGF-β dependent hepatic FoxP3+ Treg induction, thanks to their unique capacity to secrete TGF-β and to bind exogenous LAP/TGF-β to their membrane through the anchor molecule GARP. By this mechanism, LSEC-induced Tregs have been reported to become functional suppressor cells in a mouse model of autoimmune encephalomyelitis (165). Beyond this tolerogenic role, LSECs have been recognized as a population that can efficiently stimulate naïve CD8 T cells to differentiate into a liver-primed memory T cell population providing protection and contributing to clearance of viral infections (8, 166).

Also HSCs can function as tolerogenic regulators in the liver, by enhancing TGF-β-dependent Treg generation and inhibiting TGF-β dependent Th17 differentiation, via a retinoic acid (RA)-mediated mechanism (167). HSCs have been associated with the production of TGF-β and all-*trans* retinoic acid (ATRA), both important for Treg differentiation (168). In particular, generation of FoxP3+ Tregs have been reported to occur in the presence of DCs and low concentrations of TGF-β in an ATRA-dependent manner, since blocking experiments of RA-RA receptor interaction inhibited HSC-induced FoxP3 expression (169). Furthermore, a recent report indicates that HSCs from HBV patients with advanced liver fibrosis play an important role in modulating the intrahepatic Treg population via a PGE2/EP2 and EP4 pathway (170).

### T Cell Killing by NK Cells

NK cells are highly enriched within the liver where they exert a key role in anti-viral control but can also exert a regulatory effect with possible inhibition of adaptive immune responses (32, 34) (Figure 2B). Initial studies performed in LCMV-infected mice demonstrated that T cells are susceptible to NK-cell-mediated killing (171, 172). The elimination of activated T lymphocytes by NK cells can be mediated by NKG2D- and TRAIL-dependent mechanisms, as demonstrated *in vitro* (34, 173–175) and in LCMV infected mice *in vivo* (172). Moreover, regulation of T-cell responses by a direct perforin-dependent NK-cell-mediated elimination of CD4 T cells leading to the loss of help for CD8 T cells was observed in the same murine model of chronic viral infection (171).

In this negative modulation of T cell responses by NK cell killing, the NK cell NCR1 (NKp46)-receptor may have an important role and inhibition of NCR1 ligand expression on T cells by type I IFN signaling can protect T cells from NK cell killing allowing T cell evasion from NCR1 mediated NK cell attack (176).

In chronic HBV patients NK cells are more pathogenic than protective because defective in cytokine production but efficiently able to express cytolytic function (32, 34). Moreover, apoptosis of HBV-specific CD8 T cells up-regulating the death-inducing receptor TRAIL-R2 can be caused by TRAIL-positive NK cells in chronically infected HBV patients (34). By these mechanisms NK cells can deeply impair T cell responses, protecting the host from fatal immunopathology during viral infections but also diminishing the anti-viral activity of effector T cells, thereby contributing to the exhausted T cell phenotype of chronic infections. Thus, therapeutic strategies aimed at promoting the protective over the pathogenic and inhibitory effects of NK cells may represent potential options to treat chronic HBV infection.

## Perspectives for Novel Therapeutic Strategies

Immune reconstitution of functionally efficient T cell responses can be crucial for the cure of chronic HBV infection. An efficient strategy to improve the HBV-specific T cell function should probably target multiple mechanisms, because the protective anti-viral T cell response is affected by multiple factors simultaneously during chronic infections. Decline of antigen can allow correction of a single inhibitory mechanism and is expected for this reason to be insufficient alone for successful immune restoration. It thus represents only a part of the overall functional reconstitution strategy which should be put in place to cure HBV infection. Instead of currently available NUCs, which are poorly effective in diminishing HBV antigen concentrations, new and more efficient antivirals should be employed in the future, once clinically available, to reduce more rapidly and more efficiently the antigen load and the number of infected hepatocytes. They include RNA interference (RNAi) molecules, which can directly target HBV mRNAs, allowing reduction in HBsAg serum titers (177), HBV capsid assembly inhibitors and nucleic acid polymers (NAPs), as HBsAg release inhibitors (178–180). Since decline of antigen alone is expected to be insufficient, additional mechanisms should be targeted to further improve T cell responsiveness. Immune checkpoint blockade represents a possibility, but also metabolic and epigenetic modulations are very promising. In addition, recent studies performed in a transgenic mouse model of neonatal HBV infection, which closely recapitulates the immunological events occurring in the early immune tolerant phase of chronic HBV infection, indicate that IL-2, rather than checkpoint blockade, can improve defective T cell responses (74). As predictable, IL-2 treatment induced functional improvement only of HBV-specific CD8 T cells from immune-tolerant, but not immune-active chronic HBV patients.

In the perspective of a therapeutic correction of T cell exhaustion, a crucial limitation is represented by the incomplete information we have on level and quality of T cell functional reconstitution which is needed to achieve control of chronic HBV infection.

In order to address this issue, the optimal comparator to define level and quality of the T cell response to be restored for control of infection would be represented by patients able to resolve an acute infection spontaneously. However, one major limitation to the definition of the immune parameters in these subjects is represented by the heterogeneity of T cell reactivities in relation to the time elapsed from the initial exposure to the virus, which is generally undefined. Certainly the study of adult-onset infections with known duration evolving either toward resolution or chronicization would be crucial for the identification of T cell features associated to virus control. Nonetheless, the paucity of clinically overt acute HBV infections nowadays and the much greater rarity of those chronically evolving make such a study hardly feasible. In addition, difficult recruitment of a significant control patient cohort is further compounded by technical curbs due to patients’ HLA heterogeneity, as most of the studied T cell epitope specificities are mainly limited to the HLA-A^∗^02 restriction, which is only prevalent in the Caucasian population.

Even more important, still unknown, is whether the maximal improvement of T cell responses that a given chronic patient can reach after long-term exposure to HBV and its antigens is sufficient for complete and durable HBV cure. In the lack of definitive data ruling out the possibility that T cell functional defects derived from decades of T cell/virus interplay are only partially and insufficiently reversible by therapeutic T cell correction strategies, an alternative possibility to overcome T cell exhaustion is represented by the *in vitro* generation and expansion of functionally efficient, genetically engineered HBV-specific T cells for adoptive transfer to chronically infected patients. To this purpose, autologous T cells of HBV-unrelated specificity have been engineered to express HBV-specific TCR able to recognize HLA/peptide complexes in a HLA restricted manner (TCR-redirected T cells) or chimeric antigen receptors (CAR) composed of synthetic antibody fragments, combined with costimulatory domains, such as the CD28 and the CD3 zeta molecules, that allow the receptor to recognize viral antigens on infected cells in a HLA independent way, without the need of antigen processing (181–184). Thus, an important advantage of CAR T cells over TCR-redirected T cells is that CAR T cells can be used in all patients, irrespective of their HLA profile, while TCR-redirected T cells can only be used in patients with the appropriate HLA haplotype and can recognize only individual epitopes generated by intracellular antigen processing.

Potential problems related to these T cell transfer approaches are the possible risk of severe liver damage and the possible inhibitory effect on transferred effector T cells of the tolerogenic liver environment. To reduce cell lifespan and limit the risk of uncontrolled proliferation with progressive liver damage, a transient expression of the modified TCR has been developed by mRNA electroporation (185). Moreover, combination of checkpoint blockade and CAR T cell therapy (186), as well as shRNA knockdown of PD-1 in TCR-redirected T cells (187) have been used to prevent immunosuppressive mechanisms mediated by inhibitory co-receptors (188). In addition, CAR T cells have recently been further engineered by over-expressing the canonical AP-1 transcription factor c-Jun to render them resistant to exhaustion. These cells showed different chromatin accessibility, enhanced expansion and functional capacity, and improved anti-tumor activity in five different tumor mouse models *in vivo* (189).

In summary, T cell modulation remains a promising therapeutic strategy to cure chronic HBV infection, but the proof of concept that it can actually work in this clinical context still remains to be provided. A number of different elements contribute to the complexity of its practical application: first of all, the still partial knowledge of the immunological correlates of protection in HBV infection. The functional efficiency of HBV-specific T cell responses is certainly essential for a final and persistent control of infection, but we don’t know whether a selective improvement of the T cell function can be sufficient to cure infection. This uncertainty raises the issue of which other effectors of the immune system should be modulated in combination with a functional T cell reconstitution, in consideration of the evidence that also intracellular innate responses and NK cells are defective in chronic HBV infection (32, 190). This opens in turn a series of additional questions. In particular, the number of different therapeutic interventions to be used simultaneously or sequentially in order to restore a functional immune system, considering that T cell dysfunction *per se* is multifactorial and may require for its correction the coordinated application of different therapeutic approaches. This is particularly relevant in consideration of the fact that available therapies are easy to take and almost totally free of side effects and that chronic HBV patients under therapy feel absolutely well (191). This implies that new therapies should be easy to take, free of important side effects, and highly effective against the virus in a short time. In light of these considerations, all different strategies exposing the patient to the risk of a strong stimulation of T cells of HBV-unrelated specificity (i.e., epigenetic therapies, checkpoint inhibition) or of extensive and severe liver damage (i.e., therapeutic vaccines) or the combination of different approaches may be ethically problematic. This concern may also apply to the adoptive T cell transfer of genetically engineered T cells which is also technically very complex for a wide application in the clinical practice.

## Final Remarks

A number of specific drugs able to intervene on the different steps of the HBV life cycle and on different mechanisms involved in the pathogenesis of T cell exhaustion are now available and under clinical evaluation. In a first step of therapy, new direct anti-viral drugs acting on HBV replication, antigen production and liver inflammation may be needed to make immune therapies more effective, because their efficacy in improving the T cell function is believed to be affected by the negative regulatory mechanisms triggered by the exposure to high antigen quantities and by the environment of the chronically inflamed liver. This initial step of anti-viral therapy may be also essential to diminish the risk of severe liver pathology which may follow a strong activation of anti-viral effector T cells induced when the percentage of infected liver cells is elevated. Assuming that decline of antigen and control of liver inflammation may be insufficient for optimal restoration of anti-viral T cell functions, the possible choice for a second step of therapy to further overcome T cell exhaustion is in principle between checkpoint blockade, metabolic correction and epigenetic modulation strategies. On one hand, correction of mitochondrial and proteasomal defects in chronic HBV infection by the use of mitochondrial targeted anti-oxidants and polyphenolic compounds seems to be comparably or even more efficient than PD-1/PD-L1 blockade in functional T cell restoration *in vitro* with very limited effects on T cells of HBV-unrelated specificity (192). On the other hand, epigenetic modulators are expected to be highly effective on T cell responses given the massive transcriptional down-regulation detected in exhausted HBV-specific CD8 cells (36), but their use *in vivo* may be precluded by their potentially severe side effects.

Once T cell exhaustion is maximally corrected and responsiveness to antigen stimulation is optimally reacquired, effector T cell responses will likely need to be boosted in a third step of therapy by vaccines containing not only HBsAg and HBcAg but also polymerase, in consideration of its high immunogenicity and the severe level of exhaustion of pol-specific CD8 T cells reported in chronic HBV infection (39, 40).

Finally, an important goal for future research will be also to develop reliable predictors of response to immune therapies to be used in individual patients. Indeed, it is becoming increasingly clear that chronic patients are a heterogeneous population with variable levels of T cell functionality, which can probably confer propensity to respond more or less efficiently to immune modulation (37, 38). Identification of cell-mediated immunologic profiles predictive of response to immune modulation will be pivotal for the success of immune therapies because we can predict that only a proportion of chronic HBV patients will take advantage from immune modulation.

Thus, the possible scenario that recent research results allow to depict for a near future would be a therapy for chronic hepatitis B with new direct anti-viral compounds started after an immunological characterization of individual treated patients to predict their likelihood of responsiveness to immune therapies aimed at functional T cell reconstitution. Only patients predicted to be immunologically responsive will receive this second step of therapy based on immune modulation (i.e., metabolic or checkpoint modulators) and therapeutic vaccination, either sequentially or simultaneously.

## Author Contributions

PF, CB, and VB: design and writing of the manuscript. MR, GA, and DL: contribution to figure drawing. IM, AV, and AZ: contribution to the selection of the references to quote. AP and GM: discussion of the concepts to be introduced in the text. CF: critical revision of text and figures.

## Conflict of Interest

The authors declare that the research was conducted in the absence of any commercial or financial relationships that could be construed as a potential conflict of interest.
